# A Minimal Hybrid Sterility Genome Assembled by Chromosome Swapping Between Mouse Subspecies (*Mus musculus*)

**DOI:** 10.1093/molbev/msae211

**Published:** 2024-10-15

**Authors:** Vladana Fotopulosova, Giordano Tanieli, Karel Fusek, Petr Jansa, Jiri Forejt

**Affiliations:** Laboratory of Epigenetic Regulations, Institute of Molecular Genetics of the Czech Academy of Sciences, Vídenska 1083, 14220 Prague 4, Czech Republic; Laboratory of Epigenetic Regulations, Institute of Molecular Genetics of the Czech Academy of Sciences, Vídenska 1083, 14220 Prague 4, Czech Republic; Laboratory of Epigenetic Regulations, Institute of Molecular Genetics of the Czech Academy of Sciences, Vídenska 1083, 14220 Prague 4, Czech Republic; Laboratory of Epigenetic Regulations, Institute of Molecular Genetics of the Czech Academy of Sciences, Vídenska 1083, 14220 Prague 4, Czech Republic; Laboratory of Epigenetic Regulations, Institute of Molecular Genetics of the Czech Academy of Sciences, Vídenska 1083, 14220 Prague 4, Czech Republic

**Keywords:** hybrid, genomes, chromosome, *Mus musculus*, meiosis, speciation

## Abstract

Hybrid sterility is a reproductive isolation barrier between diverging taxa securing the early steps of speciation. Hybrid sterility is ubiquitous in the animal and plant kingdoms, but its genetic control is poorly understood. In our previous studies, we have uncovered the sterility of hybrids between *musculus* and *domesticus* subspecies of the house mouse, which is controlled by the *Prdm9* gene, the X-linked *Hstx2* locus, and subspecific heterozygosity for genetic background. To further investigate this form of genic-driven chromosomal sterility, we constructed a simplified hybrid sterility model within the genome of the *domesticus* subspecies by swapping *domesticus* autosomes with their homologous partners from the *musculus* subspecies. We show that the “sterility” allelic combination of *Prdm9* and *Hstx2* can be activated by a *musculus/domesticus* heterozygosity of as few as two autosomes, Chromosome 17 (Chr 17) and Chr 18 and is further enhanced when another heterosubspecific autosomal pair is present, whereas it has no effect on meiotic progression in the pure *domesticus* genome. In addition, we identify a new X-linked hybrid sterility locus, *Hstx3*, at the centromeric end of Chr X, which modulates the incompatibility between *Prdm9* and *Hstx2*. These results further support our concept of chromosomal hybrid sterility based on evolutionarily accumulated divergence between homologous sequences. Based on these and previous results, we believe that future studies should include more information on the mutual recognition of homologous chromosomes at or before the first meiotic prophase in interspecific hybrids, as this may serve as a general reproductive isolation checkpoint in mice and other species.

## Introduction

According to the biological species concept (Mayr 1963), a species is defined as a group of populations separated from other populations by reproductive isolation. Hybrid sterility is one of the earliest mechanisms of reproductive isolation that limits or completely blocks free gene flow between incipient species, thereby catalyzing their divergence. Hybrid sterility as a universal phenomenon occurs in all eukaryotic species, from fungi to plants to mammals (Coyne and Orr 2004; Presgraves and Meiklejohn 2021; Forejt and Jansa 2023). Two rules of reproductive isolation universal to all sexually reproducing organisms (Haldane 1922; Coyne 2018; Xiong et al. 2023) are still incompletely understood. According to Haldane's rule: “If in the F1 offspring of two different animal races one sex is absent, rare, or sterile, that sex is the heterozygous [heterogametic] sex,” while “The Large X Effect” states that genes responsible for hybrid sterility are more likely to be found on the Chr X than on the autosomes (Coyne and Orr 2004; Presgraves 2018). How could evolutionary forces select for infertility, the trait that handicaps hybrids, puzzled Darwin already (Darwin 1859). The solution was offered by explaining hybrid sterility as a consequence of the independent evolution of mutually interacting genes causing their incompatibility in hybrids, known as the Dobzhansky–Muller (DM) incompatibility (Dobzhansky 1951).

Our understanding of the genetic architecture of hybrid sterility has been based mainly on the studies in *Drosophila* species. The consensus model posits the threshold effects of mutually interchangeable small-effect genes with complex epistatic interactions and the disproportionally large involvement of the Chr X (Coyne and Orr 2004; Presgraves and Meiklejohn 2021). The role of genes with major effects (*OdsH*, *JYalpha*, and *Overdrive*) is considered “large-effect outliers” in this model (Presgraves and Meiklejohn 2021).

In contrast, a series of studies in the house mouse subspecies, *Mus musculus musculus* and *Mus musculus domesticus* (hereafter *musculus* and *domesticus*) provided a simpler model based on the genic regulation of chromosomal incompatibilities. Unlike the largely polygenic nature of hybrid sterility in *Drosophila*, the architecture of *Prdm9*-driven hybrid sterility in mice consists of three main components, the *Prdm9* gene (Mihola et al. 2009) showing negative epistatic interaction with an unknown gene at the X-linked *Hstx2* locus (Bhattacharyya et al. 2014; Lustyk et al. 2019) and the *musculus/domesticus* F1 heterozygosity preventing proper meiotic synapsis of evolutionarily diverged homologous chromosomes (Forejt et al. 2021; Forejt and Jansa 2023). Complete meiotic arrest of hybrids is restricted to a particular allelic combination of *Prdm9* and *Hstx2* hybrid sterility genes. Only (*musculus* × *domesticus*) F1 males heterozygous for the *dom2* (*domesticus* origin) and *msc1* (*musculus* origin) alleles of *Prdm9* and carrying the *PWD* (*musculus*) allele of the *Hstx2* locus are completely infertile (Mukaj et al. 2020; AbuAlia et al. 2024).

Sterile hybrids are vigorous but show arrest of primary spermatocytes at mid-late pachytene stage, resulting in severe reduction of testicular weight and absence of spermatozoa. A characteristic meiotic finding is incomplete synapsis of homologous chromosomes at the first meiotic prophase. Approximately 80% of pachynemas show one or more autosomal pairs asynapsed and marked by phosphorylated histone H2AX (γH2AX) and HORMAD2 protein. Further studies have shown that asynapsis is chromosome-autonomous, dependent on interaction between heterosubspecific (*musculus/domesticus*) autosomes (Gregorova et al. 2008; Bhattacharyya et al. 2013). Consubspecific (*musculus/musculus*) homologous chromosomes in heterosubspecific hybrids synapse normally, as do the pairs with a consubspecific interval of ∼30 Mb or more (Bhattacharyya et al. 2013; Gregorova et al. 2018; Valiskova et al. 2022). Another meiotic feature associated with hybrid sterility is abnormal sex body formation accompanied by disruption of transcriptional silencing of sex chromosomes in the first meiotic prophase (Homolka et al. 2007; Bhattacharyya et al. 2013), known as meiotic sex chromosome inactivation (MSCI; Mahadevaiah et al. 2008; Cloutier and Turner 2010).

The PR domain containing 9 (*Prdm9*) is the first hybrid sterility gene identified in vertebrates (Mihola et al. 2009). PRDM9 activates meiotic recombination hot spots (Baudat et al. 2010; Myers et al. 2010; Parvanov et al. 2010) by trimethylation of lysine 4 and lysine 36 at histone 3 meiotic recombination sites and makes them accessible to SPO11 topoisomerase-like protein to generate DNA double-strand breaks (DSBs). The DSB repair by homologous recombination using the DNA sequence of the homologous chromatids as a template (Arter and Keeney 2023) is a prerequisite of homologous chromosome synapsis at the first meiotic prophase and their proper segregation into gametes.

The zinc finger array of the PRDM9 histone methyltransferase is highly polymorphic and each *Prdm9* allele determines its own allele-specific binding motif, thus defining the polymorphism of the recombination hot spots (Buard et al. 2014; Vara et al. 2019; Mukaj et al. 2020; AbuAlia et al. 2024). A characteristic feature of PRDM9 hot spots is their continuous evolutionary erosion by gene conversion (Baker et al. 2015). Independent erosion of a fraction of the *domesticus* hot spots, specified by the *Prdm9^dom2^* allele in the B6 mouse strain, and the *musculus* hot spots, specified by the *msc1* allele in the PWD mouse strain, results in their heterozygosity (asymmetry) in (*musculus*^PWD^ × *domesticus*^B6^)F1 hybrids. As a result, a fraction of intact PWD hot spots is preserved on B6 homologs, while being erased, damaged, or inaccessible on their own PWD homologous chromosome, and vice versa for B6 hot spots (Davies et al. 2016, 2021; Forejt 2016; Smagulova et al. 2016). It was proposed that such asymmetric hot spots are difficult or impossible to repair by homologous recombination (Davies et al. 2016), which results in impaired synapsis of heterosubspecific homologs and meiotic arrest at the first meiotic prophase (Bhattacharyya et al. 2013; Gregorova et al. 2018; Forejt et al. 2021). Another, not mutually exclusive explanation for hybrid sterility, points to the occurrence of PRDM9-independent, default recombination hot spots, which are observed at higher frequencies in (*musculus*^PWD^ × *domesticus*^B6^)F1 males (Smagulova et al. 2016). These recombination hot spots occur in sterile *Prdm9* null mutants of both sexes and, in contrast to *Prdm9* hot spots, are predominantly located at gene promoters and enhancers (Brick et al. 2012; Mihola et al. 2019).

The involvement of the (*musculus*^PWD^ × *domesticus*^B6^)F1 hybrid genome in *Prdm9*-driven hybrid sterility is mandatory as shown by the fertility of males carrying the same “sterility” allelic combination of *Prdm9^msc1/dom2^* and *Hstx2^PWD^* in the otherwise *domesticus* C57BL/6J (abbreviated B6) genome (Bhattacharyya et al. 2013). Previously, we sought to determine the minimum proportion of the *musculus/musculus* consubspecific genome required to rescue hybrid sterility. To do it, we constructed genotypes in which all chromosomes were heterosubspecific (*musculus*/*domesticus*) except for one (Bhattacharyya et al. 2013) or up to four, asynapsis most sensitive autosomal pairs (Gregorova et al. 2018) that were made consubspecific (*musculus*/*musculus*; Gregorova et al. 2018). This showed that fertility of male hybrids could be partially restored by the presence of two or more consubspecific autosomes. Here, for the first time, a reverse approach was taken by asking what the minimum number of asynapsis most sensitive heterosubspecific autosomes is to activate the *Prdm9–Hstx2* DM incompatibility. To do this, we modified the genome of B6 laboratory (*domesticus*) mice by substituting their *domesticus* chromosomes for *musculus* homologous chromosomes from PWD strain. The results show that the presence of two or three of the smallest heterospecific autosomal *musculus^PWD^*/*domesticus^B6^* pairs (chromosomes [Chrs] 17, 18, and 19) within the *domesticus* genome is sufficient to activate *Prdm9–Hstx2* DM incompatibility and significantly reduce male fertility. The implications of these results for the concept of genic versus chromosomal hybrid sterility are discussed.

## Results

To monitor the effect of the *musculus/domesticus* heterozygosity necessary to support *Prdm9^msc1/dom2^–Hstx2^PWD^* DM incompatibility, we sequentially replaced *domesticus* Chrs 17, 18, and 19 in B6 genome with their PWD *musculus* homologs, using the C57BL/6-chr#^PWD/J/ForeJ^ chromosome substitution (consomic) strains (Gregorova et al. 2008), hereafter abbreviated as D#, where # is the chromosome number of a PWD chromosome transferred to the B6 background. These small autosomes were chosen because they are most likely to fail to synapse in hybrid males (Gregorova et al. 2018). When transferring the PWD chromosomes into the B6 genome, the “sterility” *Prdm9^msc1/dom2^–Hstx2^PWD^* allelic combination was kept constant in all constructed genotypes. The fertility parameters were the relative weight of paired testes (weight of paired testes in milligrams [TW]/body weight [BW] in grams) and the total number of sperm in the epididymis (in millions). For statistical processing, the sperm counts were normalized as Log_10_(SC + 1) (hereafter abbreviated LogSC), where SC is the number of sperm in millions.

Since impaired meiotic synapsis of heterospecific autosomal pairs (Bhattacharyya et al. 2013; Forejt and Jansa 2023) is inherent to *Prdm9*-driven F1 hybrid sterility, we determined the frequency of pachynemas with one or more asynapsed autosomes on meiotic spreads. The chromosome axes of the synaptonemal complexes were visualized by immunolabeling the SYCP3 protein, and the presence of HORMAD 2 protein signaled unpaired univalents and XY chromosomes. Identification of specific autosomes (Chrs 17, 18, and 19) was performed by fluorescence in situ hybridization (FISH) microscopy (see “Materials and Methods”). The experiments were performed in two steps, designated crosses 1 and 2 as shown below. In cross 1, we swapped Chr 17^PWD^ (including *Prdm9^msc1^* allele), Chr 18^PWD^, and Chr X1s (including *Hstx2^PWD^*) with their B6 homologs in the *domesticus* B6 genome. After observing a significant decrease on the fertility parameters of the cross 1 males, we added Chr 19^PWD^ in cross 2 in anticipation of enhancing its transgressive influence on male fertility.

### Cross 1: Fertility Parameters and Chromosome Synapsis in *domesticus* Males Heterozygous for Two *musculus* Chromosomes

**Fig. 1.**
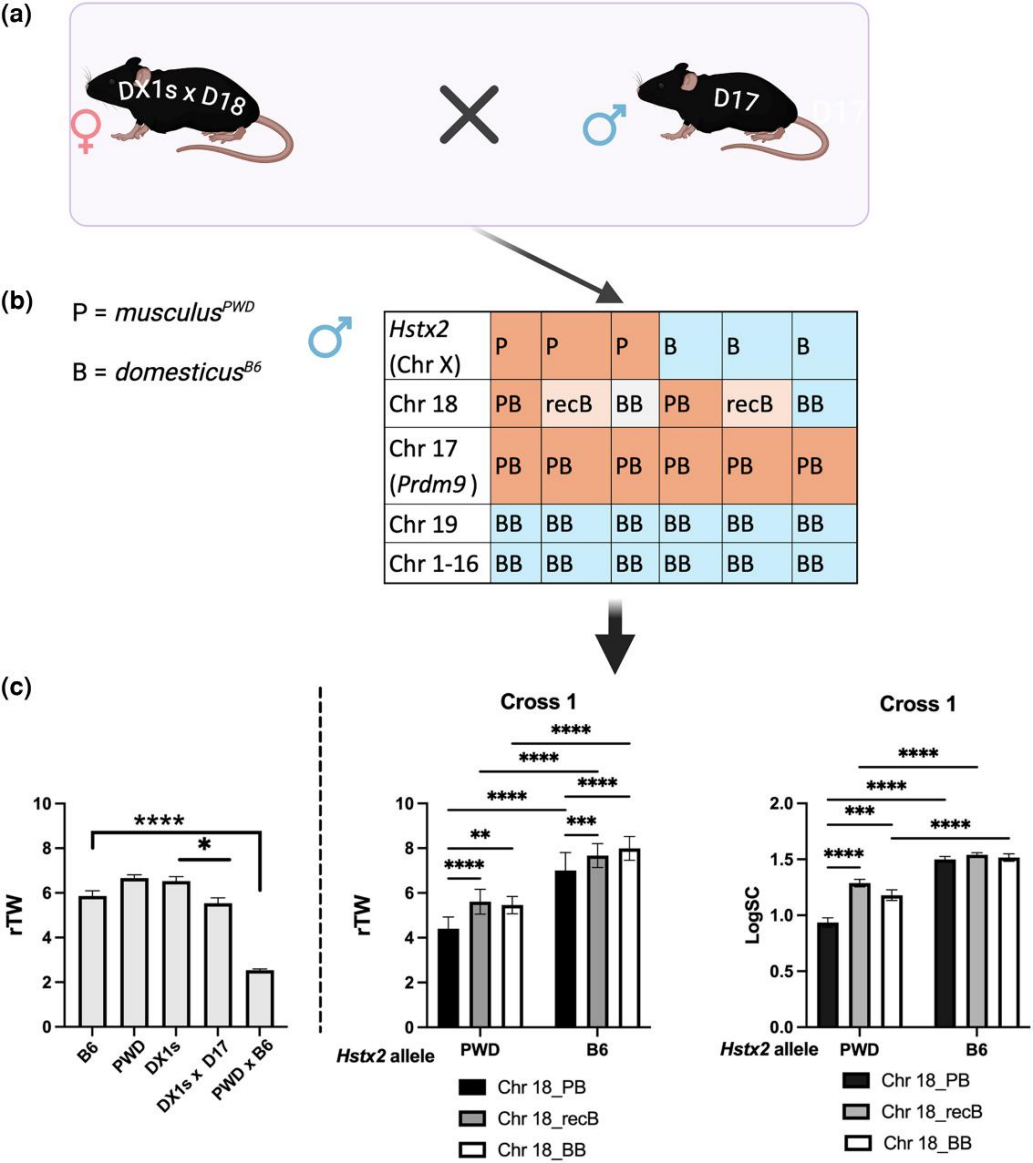
Construction of genotypes and fertility phenotypes of cross 1 males. a) The cross 1 males are the offspring of an F1 hybrid female between B6.PWD-Chr X1s and B6.PWD-Chr 18 consomic strains (DX1s and D18) and a B6.PWD-Chr 17 (D17) consomic male (see supplementary fig. S1, Supplementary Material online for details). b) The male progeny segregates six genotypes, combinations of Chr 18 heterozygosity and PWD (P) or B6 (abbreviated B) allele of *Hstx2*. All cross 1 males carry “sterility” *Prdm9^msc1/dom2^* allelic combination on Chr 17. RecB refers to recombinant maternal P/B or B/P (centromere/telomere) Chr 18 and paternal B6 (B) copy. For the localization of crossovers on maternal Chr 18 (see supplementary table S1, Supplementary Material online). c) rTW and normalized sperm count (LogSC) in cross 1 males. Parental strains and *Prdm9^msc1/dom2^*, *Hstx2P^WD^* hybrids on B6 and PWD × B6 genetic background are included for comparison. See supplementary table S1, Supplementary Material online for genotype and fertility parameters of each cross 1 male. Only significant differences (two-way ANOVA, Tukay’s post hoc) are shown (*P < 0.05, **P < 0.01, ***P < 0.001, and ****P < 0.0001). Image created with BioRender.

In cross 1, the females of C57BL/6-Chr X1.s^PWD^ consomic strain (hereafter abbreviated as DX1s), carrying the centromeric 69.21 Mb of PWD sequence on Chr X, including *Hstx2^PWD^* locus (Balcova et al. 2016), were crossed to males of the consomic strain D18 (for full genotypes see supplementary fig. S1a and b, Supplementary Material online; Gregorova et al. 2008). The resulting F1 hybrid females were then crossed to D17 males (Fig. 1a). A total of 100 male progeny of cross 1 (supplementary table S1, Supplementary Material online) segregated three Chr 18 genotypes, nonrecombinant pair of PWD/B6 or B6/B6 homologous chromosome, hereafter referred as PB and BB, and the recombinant recB pair (Fig. 1b), including recombinant paternal Chr 18 with quasi random distribution of crossovers between PWD and B6 sequence (supplementary table S1, Supplementary Material online.) Since the X-linked *Hstx2* hybrid sterility gene segregated PWD and B6 alleles (P and B in Fig. 1), the cross 1 males could be grouped into six genotypes (Fig. 1b and supplementary table S1, Supplementary Material online). All 100 males carried the “sterility” combination of *msc1* (PWD) and *dom2* (B6) alleles of the *Prdm9* gene on Chr 17. The relative testes weight (rTW) in fertile parental strains, consomics, and their hybrids were included as controls for the cross 1 (Fig. 1c). Note that the “sterility” *Prdm9^msc1/dom2^–Hstx2^PWD^* genotype causes complete meiotic arrest, small testes (rTW 2.54 ± 0.22) and absence of sperm in (PWD × B6)F1 hybrids (see also Bhattacharyya et al. 2013; Lustyk et al. 2019; Valiskova et al. 2022; AbuAlia et al. 2024) but is compatible with fertility within the *domesticus* B6 genome (DX1s × D17)F1 (Fig. 1c).

We used a two-way analysis of variance (ANOVA) with *F*-test to compare mean rTW in cross 1 males with PWD or B6 allele of *Hstx2* (*F* = 321.22, *P* < 0.0001) and PB, recB, or BB Chr 18 (*F* = 22.53, *P* < 0.0001), and significant differences were determined by Tukay's post hoc multiple comparison test. The same statistical procedure was applied for LogSC. In the *Hstx2^PWD^* males, the presence of nonrecombinant heterosubspecific Chrs 17 and 18 significantly reduced both fertility parameters, rtW (mean ± standard deviation [SD]: 4.4 ± 0.53) and LogSC 0.96 ± 0.12 compared with Chr 18 recB males—rTW 5.61 ± 0.55, *P* < 0.0001 or BB males 5.46 ± 0.39, *P* = 0.0003 and LogSC Chr 18 males 1.29 ± 0.14, *P* < 0.0001 or BB males 1.18 ± 0.13, *P* = 0.0003 (Fig. 1c).

The effect of Chr 18 subspecific heterozygosity on LogSC disappeared in the presence of *Hstx2^B6^* but was still detected on rTW (Fig. 1c). As expected, subspecific heterozygosity of only two autosomes, Chrs 17 and 18, caused only partial reduction of fertility compared with complete meiotic arrest of the (PWD × B6)F1 hybrid males. As shown below, the recB and BB Chr 18 not only had a positive effect on fertility phenotypes, but also restored synapsis of this autosomal pair at the first meiotic prophase.

**Fig. 2.**
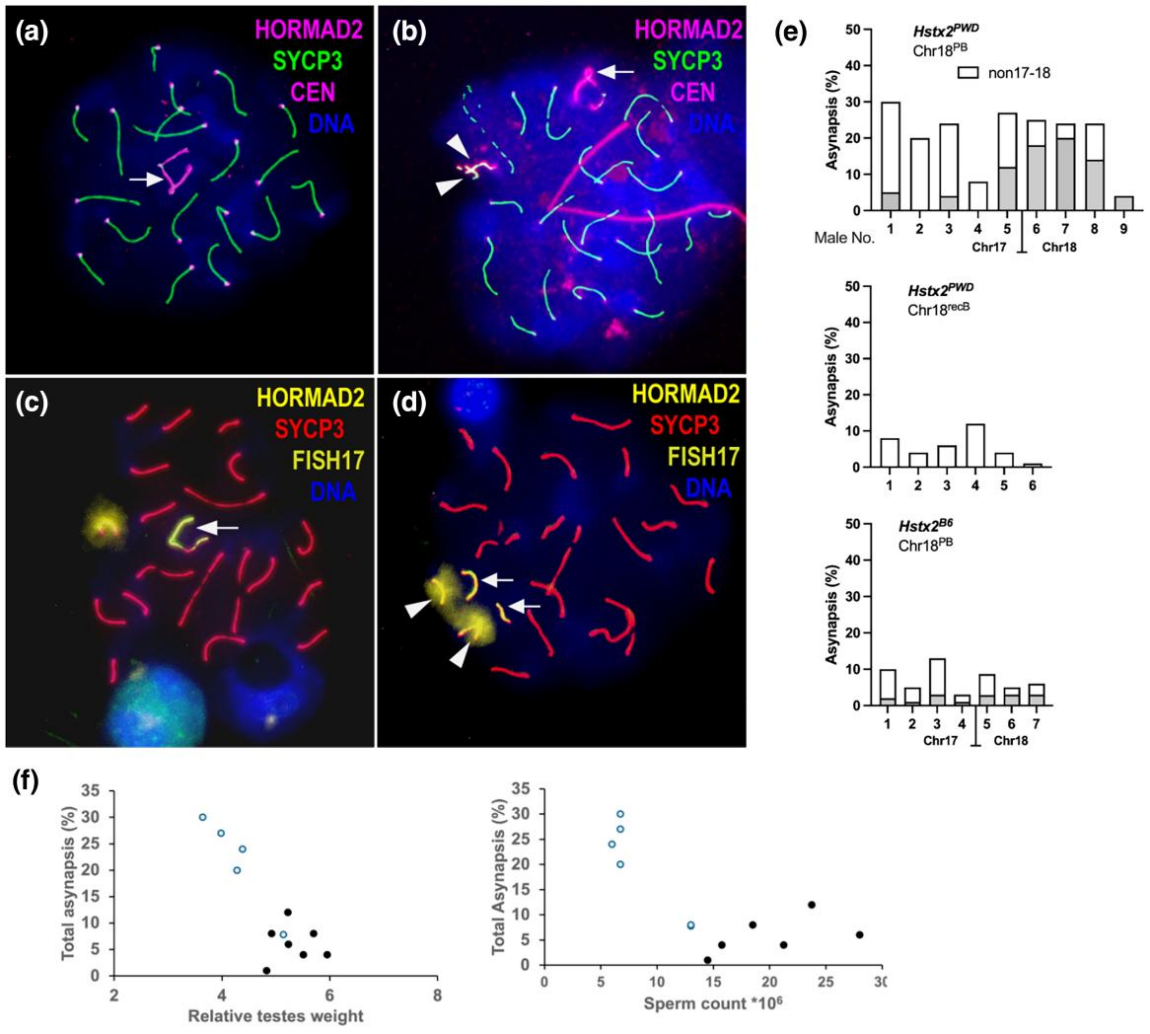
Asynapsis rate of Chrs 17 and 18 in males of cross 1. a, b) Immunofluorescence microscopy on spermatocyte spreads stained for SYCP3, HORMAD2, and centromere. HORMAD2 marks asynapsed univalents (arrowheads) and X and Y chromosomes (arrow). c) FISH on chromosome 17 (cloud) of spermatocyte spreads was combined with immunofluorescence staining of SYCP3 and HORMAD2 decorated X and Y chromosomes. d) The asynapsed univalents of chromosome 17 are marked by arrowheads, and X and Y chromosomes by arrows. e) Each column represents the percentage of pachynemas (*n* = 100) with two or more autosomal univalents from a single male. The gray parts of the columns represent the proportion of pachynemas with FISH determined asynapsis of Chr 17 or 18. Identification of Chrs 17 and 18 was not performed in *Hstx2^PWD^* males with recombinant Chr 18 (shown here as Chr 18^recB^). f) Correlation of asynapsis rate with rTW and sperm count in *Hstx2^PWD^* males with nonrecombinant PB Chr 18 (blue) or with rec/B Chr 18 (black).

Impaired synapsis of homologous chromosomes and consequent meiotic arrest at the first meiotic prophase are characteristic features of *Prdm9*-driven F1 hybrid male sterility (Forejt and Jansa 2023). To determine how restricted *musculus*/*domesticu*s heterozygosity affects meiotic pairing in males of cross 1, we estimated the frequency of pachynemas with one or more asynapsed autosomal pairs (hereafter referred to as total asynapsis rate), and specifically monitored asynapsis of Chrs 17 and 18 using FISH. The asynapsis rate of each chromosome was monitored separately in different cells of each male to increase the accuracy of identification of both chromosomes. In all males of cross 1, the Chr 17 pair was composed of maternal B6 and paternal PWD nonrecombinant copies, yet the frequency of pachynemas with Chr 17 asynapsis was very low (average 5.1%). The finding agrees with the relatively low asynapsis rate (14%) of this particular chromosome in sterile PWD × B6 F1 hybrids (Gregorova et al. 2018). Asynapsis of Chr 18 was observed in males with nonrecombinant PB homologs and was dependent on the presence of *Hstx2*^PWD^ (Fig. 2a). In the presence of the *Hstx2^PWD^*, the pachynemas with nonrecombinant PB Chr 18 homologs showed a significant increase in Chr 18 asynapsis rate (average 14%) and the total asynapsis rate (average 19.9%, 95% confidence interval [CI] 12.3% to 27.4%; Fig. 2a), and a significant reduction in rTW and sperm count (see above). The *Hstx2^B6^* males with recombinant recB Chr 18 or consubspecific BB Chr 18 showed the lowest levels of total asynapsis (mean 7.36%, 95% CI 4.6% to 10.1%), absence of Chr 18 asynapsis, and fertility parameters at the level of fertile controls.

Although we could not visualize the Chrs 18 and 17 in the same cells, the results of pachytene analysis in *Hstx2^PWD^*, Chr 18 PB males (Fig. 2a) show that most, if not all, asynapsis in these males can be attributed to these two heterosubspecific chromosomes. In addition, the total asynapsis rate correlates with rTW and sperm count, and these two phenotypes separate well the *Hstx2^PWD^* males with and without nonrecombinant PB Chr 18 (Fig. 2b).

### Cross 2: Fertility Parameters and Chromosome Synapsis in *domesticus* Males Heterozygous for Three *musculus* Chromosomes

**Fig. 3.**
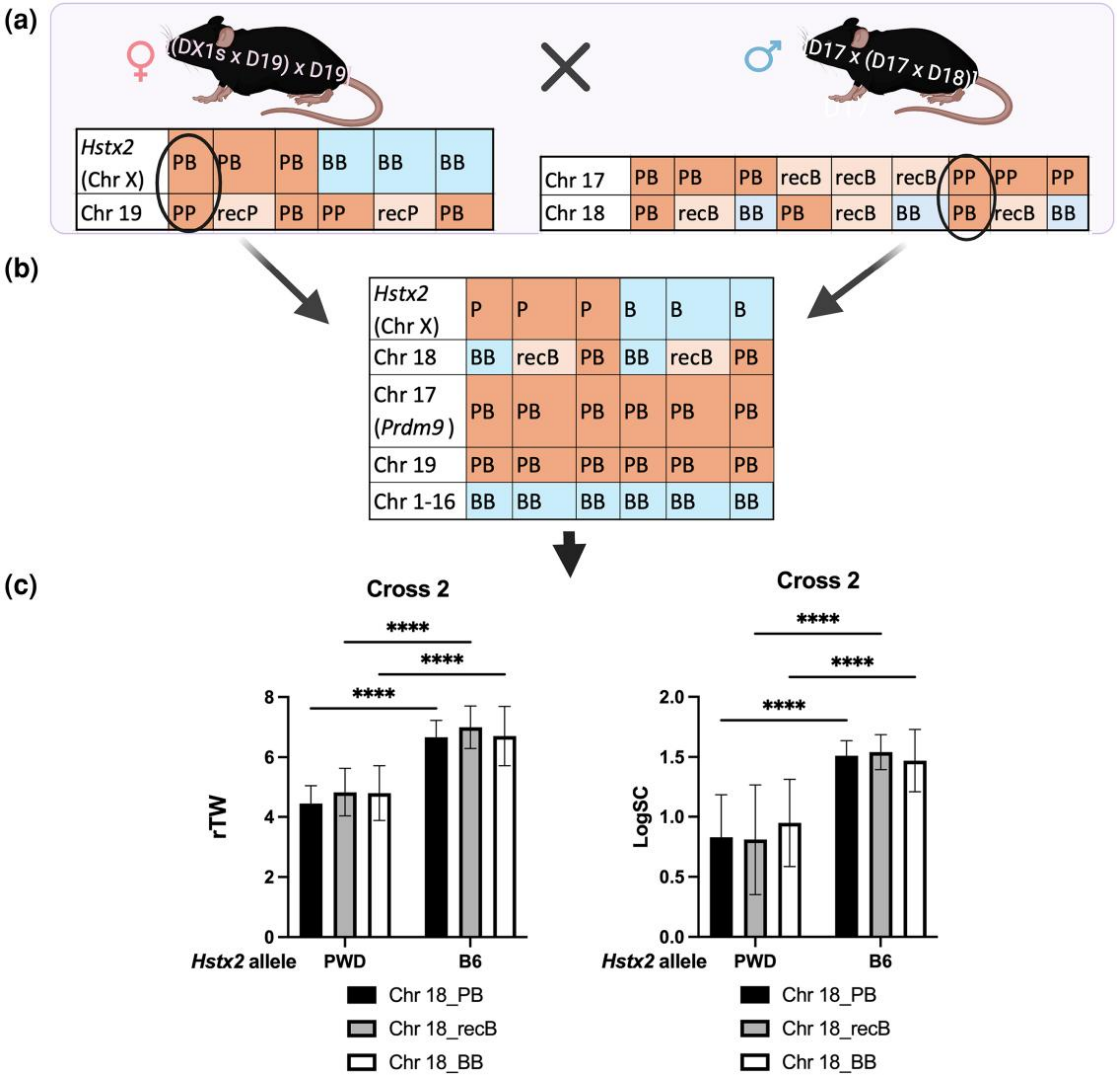
Construction of genotypes and fertility phenotypes of cross 2 males. a) To generate female parents for cross 2, the female F1 hybrids (DX1s × D19) were crossed to D19 males. Females heterozygous for *Hstx2^PWD/B6^* and homozygous for nonrecombinant Chr 19 (the circled genotype) were selected from six possible *Hstx2* and Chr 19 genotypes. The male parents were obtained from crossing (D17 × D18) F1 females to D17 males. One from nine possible genotypes was selected (circled). b) All males from cross 2 were *Prdm9^msc1/dom2^*, PWD/B6 heterosubspecific in Chrs 17 and 19. Chr 18 was either PWD/B6 or B6/B6 nonrecombinant or carried a B6/B6 consubspecific and PWD/B6 heterosubspecific parts. c) rTW and normalized sperm count (LogSC) in cross 2 males. See supplementary table S2, Supplementary Material online for genotype and fertility parameters of each cross 2 male. Only significant differences (two-way ANOVA, Tukay’s post hoc) are shown (****P < 0.0001).

**Fig. 4.**
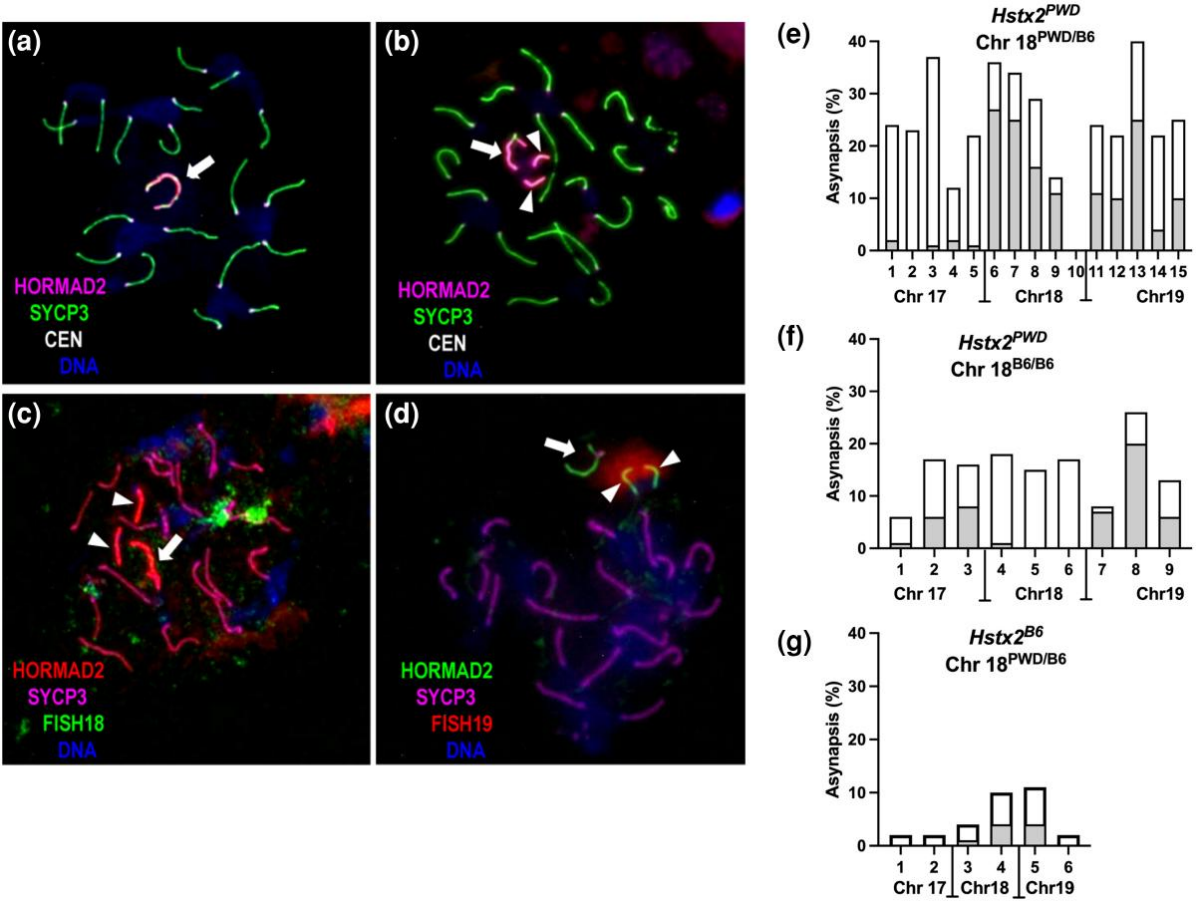
Asynapsis of Chrs 17, 18, and 19 in males of cross 2. HORMAD2 decorates X and Y chromosomes (arrow) and asynapsed univalents (arrowheads) in pachytene spreads. Chromosome axes of synaptonemal complexes stained for SYCP3 protein. a) Nineteen fully synapsed autosomal pairs in a pachytene cell with the Chr 18 BB genotype. b) A single asynapsed autosome in a Chr 18 recB cell. c) Fully synapsed Chr 18 marked by FISH using Chr 18 subcentromeric and subtelomeric DNA probe (green), a pair of univalents marked by arrowheads in Chr 18 nonrecombinant PB pachytene cell. d) Asynapsis of Chr 19 (shown in red) in a pachynema with chr 18 recB. e) Asynapsis rates of Chrs 17, 18, and 19 in *Hstx2^PWD^* males with nonrecombinant PB Chr 18 and f) in males with recB Chr18. The color-shaded parts correspond to the contribution of a specific autosome to the total asynapsis. The presence of consubspecific BB intervals in recB Chr 18 homologs completely restores the synapsis of this chromosome. g) The presence of *Hstx2^B6^* reduces total asynapsis rate even in the presence of nonrecombinant PB Chr 18.

In cross 2, a three-generation cross was set up (Fig. 3a and b and supplementary fig. S2, Supplementary Material online) to prepare males heterozygous for nonrecombinant PB homologs of Chrs 17 and 19, and as in cross 1, the males segregated *Hstx2^PWD^* and *Hstx2^B6^* alleles and three genotypes of Chr 18 (PB, BB, and recB). A total of 99 males (supplementary table S2, Supplementary Material online) were examined for rTW and LogSC, and 29 of them were examined for meiotic chromosome synapsis. The two-way ANOVA with Tukey's post hoc multiple comparison test was applied as in cross 1. Compared with cross 1, the presence of heterosubspecific Chr 19 significantly reduced LogSC in *Hstx2^PWD^* males of all three Chr 18 genotypes, with mean ± SD—0.83 ± 0.35, 0.81 ± 0.46, and 0.95 ± 0.36 for PB, recB, and BB Chr 18 genotypes, respectively, without significantly reducing rTW (Fig. 3b) (supplementary fig. S3, Supplementary Material online). The addition of Chr 19 to the heterosubspecific Chrs 17 and 18 led to an increase in the total asynapsis rate estimated by the presence of one or more asynapsed autosomes (Fig. 4a to d). The asynapsis reached 26.7% (95% CI 21.5 to 31.9) in the presence of nonrecombinant PB Chr 18 (Fig. 4e), but total asynapsis decreased when Chr 18 was BB consubpecific to 15.11% (95% CI 10.6 to 19.6; Fig. 4f) and total asynapsis further decreased to 5.5% of pachynemas in the presence of *Hstx2^B6^* (Fig. 4g), consistent with the increase in fertility parameters (Fig. 3b).

The effect of age and genetic background has been reported in the F1 male progeny of *musculus^PWK^* females and *domesticus* males of several classical laboratory inbred strains (Flachs et al. 2014; Widmayer et al. 2020). We studied the age effect on the fertility parameters in forty cross 2^Aged^ males aged 27 to 42 weeks (supplementary table S3, Supplementary Material online). Relative testicular weight was slightly reduced in aged *Hstx2^PWD^* males with the Chr 18 PB genotype (mean ± SD: 4.22 ± 0.36) compared with Chr 18 recB (4.90 ± 0.70) or BB (4.81 ± 0.73) (*P* = 0.0108). The genotype of Chr 18 had no effect on sperm count, in contrast to the *Hstx2^PWD^*, which reduced rTW and sperm count in aged males (supplementary fig. S4a, Supplementary Material online). The reduced asynapsis rate of Chr 18 in aged males (supplementary fig. S4b, Supplementary Material online) agrees with the loss of correlation between subspecific Chr 18 heterozygosity and fertility traits. Compared with males of the same genotype at 13 weeks of age, no difference was found in rTW of aged males in any of the six genotypes (supplementary fig. S5a, Supplementary Material online), contrasting with a significant increase with age in the absolute number of sperms in the epididymis (supplementary fig. S5b, Supplementary Material online). The spectacular increase in sperm production without a change in testicular weight could indicate the release of a pachytene checkpoint block, but no further studies have been done in this direction.

### *Hstx3* Hybrid Sterility Locus Attenuates *Prdm9–Hstx2* Incompatibility

**Fig. 5.**
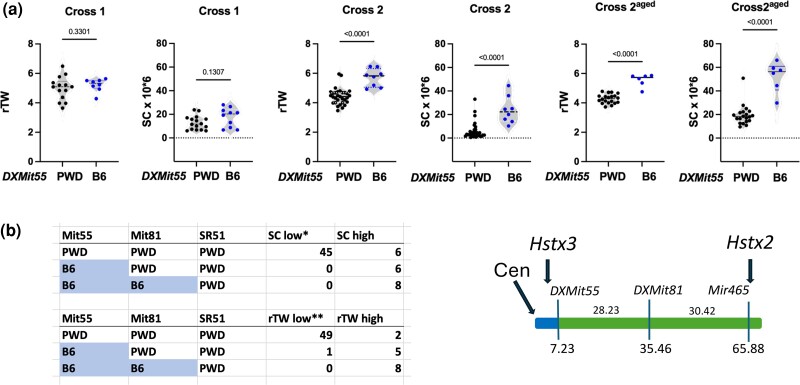
Mapping of the new hybrid sterility gene (*Hstx3)* to the 7.23 Mb centromeric interval of Chr X. a) In crosses 2 and 2^aged^, the *Hstx2^PWD^* males with the 7.23 Mb centromeric B6 sequence showed significantly increased rTW and sperm count. b) For mapping purposes, rTW and sperm counts of each male were classified as low and high. In the group with sperm count <1 × 10^7^ in cross 2 and <2.8 × 10^7^ in cross 2^aged^, there were predominantly males with nonrecombinant 65.9 Mb of PWD sequence, while males with 7.23 Mb of B6 sequence were missing. Similar asymmetry was seen between the presence of B6 sequence at the centromeric 7.23 Mb and low/high rTW. The difference was highly significant (*χ*^2^ = 43.8 and 53.60, *P* < 0.0001 for SC and rTW).

We have noticed that the variation in rTW was higher in crosses 2 and 2^aged^ than in the parental strains. Because the only segregating part of the genome of males with the same genotype of Chr 18 was the proximal region of Chr X, we scanned this 69 Mb interval for recombination between B6 and PWD sequence in search of a possible modifier. Indeed, by mapping recombinants between the centromere, microsatellite markers *DXMit55* at 7.23 Mb and *DXMit81* at 35.46 Mb and *Hstx2* locus marker *DXSR51* at 64.84 Mb (Lustyk et al. 2019), we have mapped a new hybrid sterility locus in the interval between centromere and *DXMit55*, which we have named hybrid sterility X3, *Hstx3.* The B6 allele of *Hstx3* locus significantly attenuates the sterility-promoting effect of *Hstx2^PWD^* in both sets of cross 2 (Χ 43.82, *P* < 0.0001 with 3 d.f. for rTW and 53.60, *P* < 0.0001 with 3 d.f. for sperm count). The effect of *Hstx3* was not statistically significant in cross 1, apparently because rTW and sperm count were closer to normal fertility parameters than in cross 2 (Fig. 5a and b). The 7.73 Mb interval harbors 23 protein-coding genes and 19 lncRNA, snoRNA, and miRNA genes (supplementary table S4, Supplementary Material online). The search for an *Hstx3* candidate is ongoing.

To verify the constructed genotypes, we used MiniMUGA array (Morgan et al. 2015; Sigmon et al. 2020), which discriminates over 3,500 single nucleotide polymorphism markers between PWD and B6 genome-wide, and analyzed 15 randomly selected males from all three crosses. Inspection of all 19 autosomes, X and Y sex chromosomes, and the mitochondrial genome confirmed the integrity of the genomes studied and provided no further evidence to explain variance in fertility traits (supplementary table S5, Supplementary Material online).

### Chr X Transmission Ratio Distortion

Meiotic drive is a topical issue in hybrid biology (Arora and Dumont 2022; Kitano and Yoshida 2023). We have found a significant deviation from the 50:50 segregation of the X-linked *Hstx2^PWD^* and *Hstx2B^6^* alleles in favor of *Hstx2B^6^* (supplementary tables S1 to S3, Supplementary Material online; *X*^2^(1), *n* = 239, 7.74, *P* = 0.0055). Although meiotic drive was not the subject of this study, the phenomenon should merit further attention especially since in the (PWD × B6)×B6 backcross of (Dzur-Gejdosova et al. 2012) the segregation of the *Hstx2* PWD and B6 alleles was Mendelian, 104/119.

### Pairing and Synapsis of Heterosubspecific Univalents

The failure of homologous chromosomes to synapse can be caused by their failure to meet (pairing) at the beginning of the first meiotic prophase or by their inability to match at the molecular level (synapsis). The presence of more than one asynapsed chromosome pair in (PWD × B6)F1 hybrids makes it difficult to distinguish between the inability to pair and the inability to synapse. However, in crosses 1 and 2, most pachynemas with asynapsis contain only one asynapsed, heterosubspecific pair of homologous chromosomes. In pachytene spreads, we distinguished between univalents that are widely separated within the cell or closely associated, as shown in Fig. 4b to d. Of the 325 pachynemas analyzed from three males of cross 2 and one DX1 s × D17 F1 male, 63 showed asynapsis. Of these, 37 (58.7%) showed tightly associated univalents, 5 (8%) univalents were widely separated, 9 (14.3%) pachynemas showed a partially synapsed bivalent, and the remainder (12 cells, 19%) were not suitable for analysis due to insufficient spreading. Considering the effect of spreading, which may disrupt some associations, the results strongly suggest that mutual recognition of heterosubpecific homologous chromosomes is disrupted at the level of synapsis.

## Discussion

*Prdm9-*driven hybrid male sterility is a model of reproductive isolation mechanism with a well-defined genetic architecture composed of three necessary and sufficient components: *Prdm9* gene, X-linked *Hstx2* locus and *musculus/domesticus* autosomal heterozygosity (for review, see Forejt 1996; Forejt et al. 2021; Forejt and Jansa 2023). The interallelic incompatibility of *msc1* (*musculus*) and *dom2* (*domesticus) Prdm9* alleles modulated by an X-linked *Hstx2^PWD^* (*musculus*) locus in intersubspecific F1 hybrid genome disturbs synapsis of homologous autosomes in high percentage of primary spermatocytes and causes spermatogenic arrest (Bhattacharyya et al. 2013, 2014; Gregorova et al. 2018). At the molecular level, the asymmetric binding of PRDM9 to allelic binding sites resulting in a failure to repair SPO11-programmed DNA DSBs using the homologous chromatids as a template and consequent failure of meiotic synapsis is the most likely scenario of meiotic arrest in sterile hybrids (Davies et al. 2016, 2021; Hinch et al. 2019).

Previously, we have shown that *Prdm9–Hstx2*-driven meiotic incompatibility operates in the *musculus/domesticus* F1 hybrid genetic background but not in the pure *domesticus* genome (Bhattacharyya et al. 2013; Lustyk et al. 2019; Mukaj et al. 2020). In this study, we show that to partially activate the *Prdm9–Hstx2* incompatibility in the *domesticus* genome, it is sufficient to replace two *domesticus* chromosomes (Chrs 17 and 18) with their *musculus* homologs. Sperm production can be reduced even more by adding one more heterosubspecific chromosome pair (Chr 19). With the *Prdm9^msc1/dom2^* genotype fixed in all males studied, the fertility parameters and degree of asynapsis of heterosubspecific homologous chromosomes were further dependent on the presence of the PWD allele of the X-linked *Hstx2* locus and a newly identified *Hstx3* locus.

**Fig. 6.**
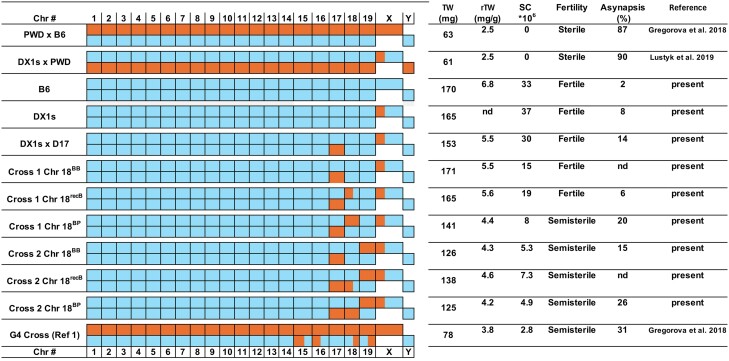
Overview of *domesticus* (B6)–*musculus* (PWD) chromosome interactions affecting male fertility parameters and meiotic synapsis. All genotypes, except B6 and DX1 mouse strains, share “sterility” allelic combination *Prdm9^msc1/dom2^–Hstx2^PWD^*.

Hybrid sterility caused by interallelic *Prdm9^msc1/do^*^m2^ incompatibility may look like an iconoclastic model of a reproductive isolation barrier. In fact, the DM incompatibility model was developed to circumvent interallelic incompatibility, which was thought to be an unlikely mechanism of reproductive isolation (Dobzhansky 1951; Coyne and Orr 2004). This paradox can be explained by the fact that the interallelic *Prdm9^msc1/dom2^* incompatibility is expressed only in the *musculus*/*domesticus* F1 hybrid background and but is lost in the context of the *domesticus* or *musculus* genome (Figs. 1 and 6;  Bhattacharyya et al. 2013; Gregorova et al. 2018).

The Chrs 15, 16, 18, and 19 are the smallest autosomes, representing 13% of the mouse genome, but in pachynemas of (PWD × B6)F1 hybrid males they are most sensitive to asynapsis (asynapsed in 20%, 23%, 30%, and 42% of pachynemas, respectively; Gregorova et al. 2018). The effect of Chr 18 nonrecombinant PB heterozygosity on fertility (Fig. 1) and asynapsis (Fig. 2) was evident in cross 1 males. The recombinant recB pairs of Chr 18 were associated with significantly higher fertility parameters and lower asynapsis rate, similar to that observed in consubspecific BB homologs. It has been shown previously that ∼27 Mb (or more) of consubspecific sequence is sufficient to completely rescue chromosome synapsis (Gregorova et al. 2018). However, when these small chromosomes were made PP consubspecific in the modified (PWD × B6)F1 hybrid males, their asynapsis disappeared and male fertility was partially restored (sperm count 4 × 10^6^ to 7 × 10^6^, see Fig. 4—source data 3 in Gregorova et al. 2018). To conclude, the *Prdm9^msc1/do^*^m2^–*Hstx2^PWD^* incompatibility can be partially abolished by consubspecific PP autosomes (Gregorova et al. 2018) as well as by consubspecific BB chromosomes (the present study), implying that only their heterosubspecific interaction activates meiotic arrest. This clearly rules out a recessive or dominant nature of the epistatic interactions between genes in the background and *Prdm9* in the PWD × B6 model of F1 hybrid sterility (Fig. 6). Taken together, the current evidence suggests that *Prdm9*-driven hybrid sterility is a special form of gene-controlled chromosomal sterility in which the primary incompatibility consists in the evolutionarily divergence of noncoding DNA sequences of homologous PRDM9-binding sites.

Another unusual feature of *Prdm9* is the interplay between biased gene conversion resulting in PRDM9 hot spot extinction, followed by positive selection favoring new PRDM9 alleles recognizing new sequence motifs, the evolutionary mechanism described by so called intragenomic Red Queen Model (Latrille et al. 2017; Genestier et al. 2024). It results in high polymorphism of recombination hot spots in population of mice, humans, and other mammalian species (Hinch et al. 2011; Kono et al. 2014; Beeson et al. 2019; Vara et al. 2019; Damm et al. 2022). Studies of the *Prdm9*-driven meiotic incompatibilities in the *musculus/domesticus* European hybrid zone are pending, but initial results from 30 wild-derived inbred strains or mice from outcrossed populations showed that only the combination of wild-derived *domesticus dom2* or *dom2*-like *Prdm9* alleles with the *msc1* or *msc1*-like *musculus* allele caused meiotic impairment (Mukaj et al. 2020; AbuAlia et al. 2024).

Studies of wild mice from the *musculus/domesticus* hybrid zone (Turner and Harr 2014; Janousek et al. 2015), as well as the QTL and eQTL mapping experiments (Vyskocilova et al. 2009; Dzur-Gejdosova et al. 2012; White et al. 2012; Phifer-Rixey and Nachman 2015; Wang et al. 2015; Payseur and Rieseberg 2016) using wild-derived mice, reported many hybrid sterility loci scattered throughout the genome with disproportional contribution of Chrs X and 17. The complexity of genetic control of hybrid sterility reported in these studies contrasts with the relatively simple genetic architecture of *Prdm9*-driven hybrid sterility. We propose two nonexclusive explanations for this apparent paradox:

- *Prdm9*-driven genomic architecture of meiotic arrest was defined for F1 hybrid sterility, but the studies in wild mice from the hybrid zone or in QTL experiments were based on backcrosses and intercrosses. In backcrosses and intercrosses, recessive genes contribute to DM incompatibility, but they cannot be involved in F1 hybrid sterility where only underdominant incompatibilities are active. Recessive incompatibilities are generally more numerous than underdominant ones (Coyne and Orr 2004). However, the role of the *Prdm9*-driven barrier must be strong, considering the meiotic rescue from the sterility of hybrids between *Mus spretus* and *domesticus* species by simply swapping the *domesticus* for the human ZnF domain in the mouse PRDM9 (Davies et al. 2021).
- We propose that some of the eQTLs may be a secondary effect of the failure of chromosome synapsis and the resulting meiotic silencing of unsynapsed chromatin (Burgoyne et al. 2009; Turner 2015), which can cause a cascade of underexpressed and overexpressed genes. Unsynapsed autosomal chromatin is also responsible for dysregulated transcriptional inactivation of X and Y chromosomes. (Homolka et al. 2007; Bhattacharyya et al. 2013; Larson et al. 2022), known as MSCI, with its own downstream effect on meiotic transcriptome interfering with normal differentiation of male germ cells (Royo et al. 2010).

## Conclusion

It is not easy to discriminate between genic and chromosomal control of hybrid sterility (Coyne and Orr 2004; Faria and Navarro 2010; Forejt and Jansa 2023), and if we ignore the meiotic consequences of synapsis failure between evolutionary diverged homologous chromosomes, we might consider the chromosomal incompatibility of heterosubspecific chromosomes as the cumulative effect of mutually interchangeable small-effect genes. But what could be the mode of action of such genes? In PWD × B6 hybrids with random stretches of consubspecific PWD/PWD sequence on several autosomal pairs the PWD/PWD allelic combination rescued fertility and the PWD/B6 combination led to sterility (Gregorova et al. 2018), whereas here the B6/B6 allelic combination was compatible with fertility and PWD/B6 was a sterility combination. This would mean that if the sterility in these two models was caused by the cumulative effect of small-effect genes, their interaction should have been underdominant rather than recessive or dominant, which is contrary to current knowledge of the behavior of hybrid sterility genes. Thus, given the underdominant nature of these elusive genes and knowing the function of the *Prdm9* gene in recombination and chromosome synapsis, the chromosomal mechanism based on failure of recognition between homologous chromosomes is the most parsimonious explanation. In this context, it is tempting to speculate that the mutual recognition of homologous chromosomes at or before meiosis may function as a universal checkpoint in reproductive isolation in other species, even including *Drosophila* males where homologs do not synapse.

## Materials and Methods

### Mice

The PWD/Ph strain (PWD) is a wild-derived mouse strain from a single pair of *musculus* mice trapped in Central Bohemia, Czech Republic, in 1972 (Gregorova and Forejt 2000). The C57BL/6J (B6) inbred strain originates from The Jackson Laboratory, Bar Harbor, ME, USA. The panel of chromosome substitution strain C57BL/6J-Chr #PWD (abbreviated here D#) was prepared in our laboratory (Gregorova et al. 2018) and is maintained in our laboratory and in The Jackson Laboratory. All mice were maintained at the Institute of Molecular Genetics in Vestec, Czech Republic, in the Specific Pathogen-Free Facility, in accordance with animal care protocols approved by the Committee on the Ethics of Animal Experiments of the Institute (No. 45/2020). The animal care obeyed the Czech Republic Act for Experimental Work with Animals (Decree No. 207/2004 Sb and Acts Nos. 246/92 Sb and 77/2004 Sb), fully compatible with the corresponding regulations and standards of the European Union (Council Directive 86/609/EEC and Appendix A of the Council of Europe Convention ETS123).

### Fertility Phenotyping

Males were euthanized by cervical dislocation at 13 weeks of age in crosses 1 and 2 and at 27 to 43 weeks of age in cross 2^aged^. BW in grams and fertility parameters, TW in milligrams, and sperm count in millions were determined as previously described (Lustyk et al. 2019). Sperm were collected from epididymis in phosphate buffered saline (PBS) solution. Counts were made using a hemocytometer chamber under a light microscope and sperm number was calculated by standard methods. Log10(SC + 1) (SC, sperm count in millions), denoted as log(SC), was used as a normalized value for sperm count in statistics calculation and graph construction.

### Genotyping and Estimation of Chromosome Subspecies Ancestry

To distinguish the *musculus^PWD^* and *domesticus^B6^* composition of Chrs 17, 18, 19, and X in parents of cross 2, and males of crosses 1 and 2 the following microsatellite loci (Marker Query Summary [jax.org] polymorphic between both mouse strains were genotyped using SSLP PCR assay as described previously; Gregorova et al. 2018; Lustyk et al. 2019): DXMit55, DXMit81, DXSX65100, DXSR51, D17Mit164, D17Mit50, D17Mit123, D18Mit66, D18Mit22, D18MIt149, D18Mit124, D18Mit7, D19Mit32, D19Mit46, and D19Mit1. The primers for PCR amplification and the position of the SSLP loci on each chromosome are shown in supplementary table S6, Supplementary Material online. Fifteen selected males were further genotyped with 3,634 subspecies-specific markers within the MiniMUGA array (Morgan et al. 2015; Sigmon et al. 2020) to confirm the integrity of the chromosomal substitution strains used.

### Immunostaining of Meiotic Spreads and Asynapsis Rate Determination

After dissection of the testes, the tunica was removed, and the tissue was placed in 500 µL of ice-cold RPMI medium. The tubules were dissociated from the connective tissue by vigorous hand shaking for ∼3 min, and allowed to sediment for 3 min, and the supernatant was removed. This step was repeated twice, and after a brief centrifugation, the tubules were macerated with small scissors. After pipetting 20 times, the solution was filtered and centrifuged at 1,500 rpm (300 ×*g*) for 5 min, and the pelleted cells were resuspended by adding 800 µL of 0.1 M sucrose containing a protease inhibitor + ethylenediaminetetraacetic acid cocktail (Roche 1836153) and incubated on ice for 12 min. The cells were then spread on slides covered with a film of fixative solution (1% paraformaldehyde in 50 mM NaBorate [pH 9.0] with 0.15% Triton X-100 with a cocktail of protease inhibitors + ethylenediaminetetraacetic acid (Roche 1836153) and incubated for 3 h in a humidified box at 4 °C. Immunostaining was performed with a modified protocol based on (Anderson et al. 1999). Briefly, slides were rinsed briefly in Millipore water, air-dried, washed once in PBS for 5 min and incubated in block buffer (PBS-1 ×, 5% goat serum, 1.5% bovine serum albumin, 0.05% TX100 with protease inhibitor (PI) cocktail 0.2 ×) for 1 h at 4 °C with a cover slip. Immunostaining was performed with the primary antibodies anti-HORMAD2 (1:500, rabbit polyclonal antibody, a gift from Attila Toth) and SYCP3 (1:100, mouse monoclonal antibody, Santa Cruz, #74569). Centromere painting was done by human antibodies from autoimmune serum (AB-Incorporated, 15-235), diluted 1:300. Primary antibodies diluted in block buffer (+PI) were added, and the slides were incubated with a cover slip overnight at 4 °C. After being washed 3× in PBS for 5 min each, secondary antibodies diluted in block buffer (+PI) were applied, and slides were incubated with a cover slip for 3 h at 4 °C. Secondary antibodies were used at 1:400 dilutions: goat anti-Mouse IgG-AlexaFluor488 (MolecularProbes, A-11029), goat anti-Rabbit IgG-AlexaFluor568 (MolecularProbes, A-11036), and goat anti-Human IgG-AlexaFluor647 (MolecularProbes, A-21445). The slides were washed 3× in PBS 5 min each and once in Millipore water, air-dried for 15 min in the dark at room temperature and mounted in Vectashield medium with DAPI and covered with a cover slip.

The images were acquired and examined using a Nikon Eclipse 400 microscope with a motorized stage control using a Plan Fluor objective, 60× (MRH00601; Nikon) and captured using a DS-QiMc monochrome CCD camera (Nikon) and the NIS-Elements programme (Nikon).

For each animal, we analyzed more than 50 pachynemas. The substages of the first meiotic prophase were identified based on the process of formation of synaptonemal complexes as described (Page et al. 2012; Lam et al. 2019). The number of asynapses per nucleus was scored in each pachynema. One asynapsis was equal to one HORMAD2 stained element, excluding XY chromosomes. The asynapsis rate was represented as the percentage of pachynemas with asynapses out of the total number of the checked pachynemas of each male. The tight association of asynapsed homologs is distinguished from a loose association when their mutual distance does not exceed the axial element length of any of them. The third form is a partial asynapsis with a synaptonemal complex forming a part of the bivalent.

### Fluorescence In Situ Hybridization

FISH was performed by using fresh or frozen testicular tissue. The meiotic spreads and the immunostaining protocol were used as described above but with different combinations of primary and secondary antibodies. As primary antibodies, mouse anti-SYCP3 (Santa Cruz #sc-74569) diluted 1:100, and rabbit anti-HORMAD2 (gift from Attila Toth) diluted 1:500 were used. For secondary antibodies, anti-Mouse IgG (Go) Alexa647 (Molecular Probes, # A-21236) diluted 1:400, anti-Rabbit AlexaFluor568 (Molecular Probes, # A-11036) diluted 1:400 (only for FISH on Chr 18) were applied. Anti-Rabbit (Go) IgG Alexa488 (Molecular Probes, # A-11034) diluted 1:400 was used only for FISH on Chrs 17 and 19. For FISH at Chr 18, probes were prepared using the Nick translation protocol (Abbott Laboratories, #07J00-001) with DNA derived from two BAC genomic clones (RP23-204B6, Chr 18: 3,767,826 to 3,981,292; RP23-79M16, Chr 18: 89,667,996 to 89,811,151 [kind gift from Daniela Moralli]). After immunostaining, the slides were washed four times in PBS for 5 min each and postfixed in 2% formaldehyde for 5 min. After another wash in PBS for 10 min, an incubation in HCl (0.1 N) for 10 min, and a wash in 2× SSC (sodium chloride sodium citrate buffer solution) for 3 min, the probes were placed on the slides under a coverslip (24 × 24) for 5 min at 85 °C. Slides sealed with nail polish were incubated overnight at 37 °C in a humidified box. The slides were then washed three times in 4×SSC at 43 °C and once briefly in deionized water and finally mounted in Vectashield medium with DAPI. For FISH on Chrs 17 and 19, the slides after immunostaining with the secondary antibodies, were washed three times in PBS and kept in PBS at 4 °C overnight. After graded dehydration in 70%, 90%, and 100% ethanol for 3 min each, the slides were air-dried at room temperature (15 to 20 min) and incubated at 65 °C for 1 h. After denaturation in 70% formamide in 0.6×SSC at 73 °C for 7 min (pH = 7.2), the slides were immersed in 70% ice-cold ethanol for 3 min and dehydrated in graded series of 70%, 90%, and 100% ethanol for 3 min each. Probes (MetaSystems Probes) were prepared according to the manufacturer's instructions (denatured at 75 °C for 5 min and renatured [preannealed] at 37 °C for 30 min to 1 h). Ten microliters of prehybridized probes were added directly to a slide (incubated for 5 min at 42 °C), a coverslip (24 × 24) was placed and sealed with rubber cement and hybridized for 72 h at 42 °C in a water-filled (saturated) chamber. After washing in 4× SSC at 42 °C (3×; 5 min each), the slides were mounted in Vectashield mounting media with DAPI. All the steps were carried out in the dark with minimum exposure to light.

### Statistics

Variances in relative weight of paired testes (rTW) and sperm count were evaluated by two-way ANOVA followed by multiple comparisons using Tukey's post hoc test. The effect of recombination between centromere and position 7.23 Mb on Chr X on fertility traits was evaluated by unpaired *t*-test for rtW and by the two-tailed Mann–Whitney *U* test for differences in sperm count. Statistical analysis was performed using GraphPad Prism Version 10.2.3, Graphpad Software, La Jolla, CA, USA.

## Supplementary Material

msae211_Supplementary_Data

## Data Availability

Mouse strains used in this work are available on request. The authors affirm that all data necessary for confirming the conclusions of the article are present within the article, figures, tables, and in the Supplementary material. The raw image data are available upon reasonable request.
