# Supplementary material for: A Minimal Hybrid Sterility Genome Assembled by Chromosome Swapping Between Mouse Subspecies (Mus musculus)
Source: Mol Biol Evol. 2024 Oct 15;41(10):msae211. doi: 10.1093/molbev/msae211 (PMC11518865; doi:10.1093/molbev/msae211)
Supplement: msae211_Supplementary_Data [file msae211_supplementary_data.zip › SUPPLEMENTARY MATERIAL.pdf]

SUPPLEMENTARY MATERIAL

A minimal hybrid sterility genome assembled by chromosome swapping between mouse subspecies (*Mus musculus*)

Authors

Vladana Fotopulosova*[1,2], Giordano Tanieli[1]*, Karel Fusek[1], Petr Jansa[1] and Jiri Forejt[1]^

Affiliations:

[1]Laboratory of Epigenetic Regulations, Institute of Molecular Genetics of the Czech Academy of Sciences, Vídenska 1083, 14220 Prague 4, Czech Republic.

[2]Current address: fotopulosova@exbio.cz

^Address for correspondence: Jiri Forejt, Laboratory of Epigenetic Regulations, Institute of Molecular Genetics of the Czech Academy of Sciences, Vídenska 1083, 14220 Prague 4, Czech Republic. E-mail: jforejt@img.cas.cz

INDEX

Supplementary Figures 3
Supplementary Tables Legend 8

Supplementary Figures

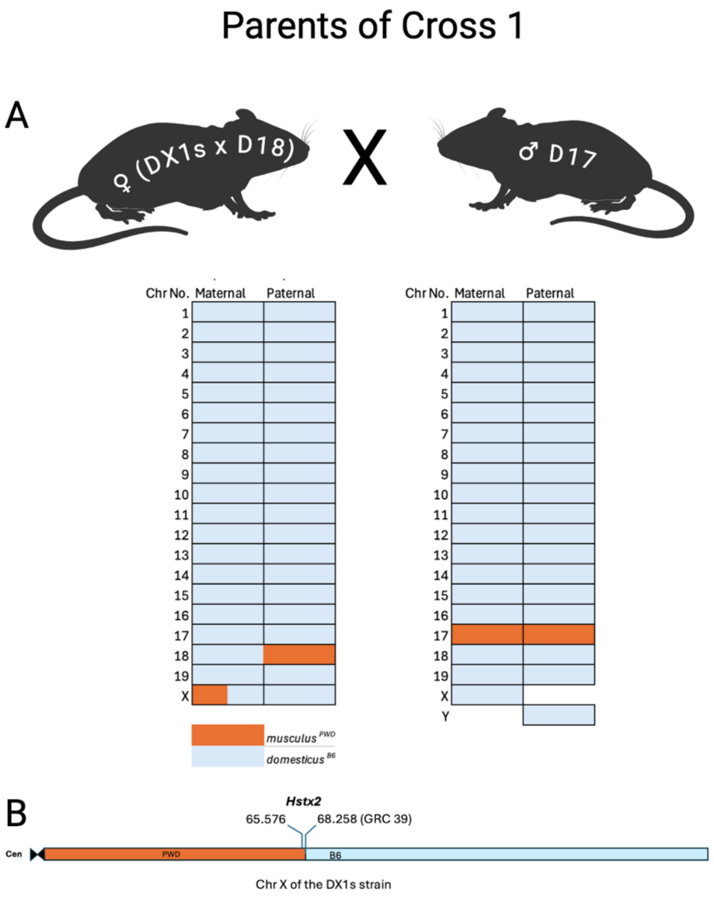

Fig. S1 | Chromosomal constitutions of the parents of Cross 1. ***A***. The female carries a maternal copy of Chr X from the DX1s consomic strain and a paternal copy of Chr 18 from the D17 consomic strain; the male parent has both copies of Chr 17 of PWD origin. The chromosomes of B6 (*domesticus*) origin are blue, the PWD (*musculus*) chromosomes are orange. ***B.*** Chr X of the DX1s consomic strain carries 68.3 Mb of PWD sequence at the centromeric end. The *Hstx2* locus occupies the distal 2.68 Mb of this interval

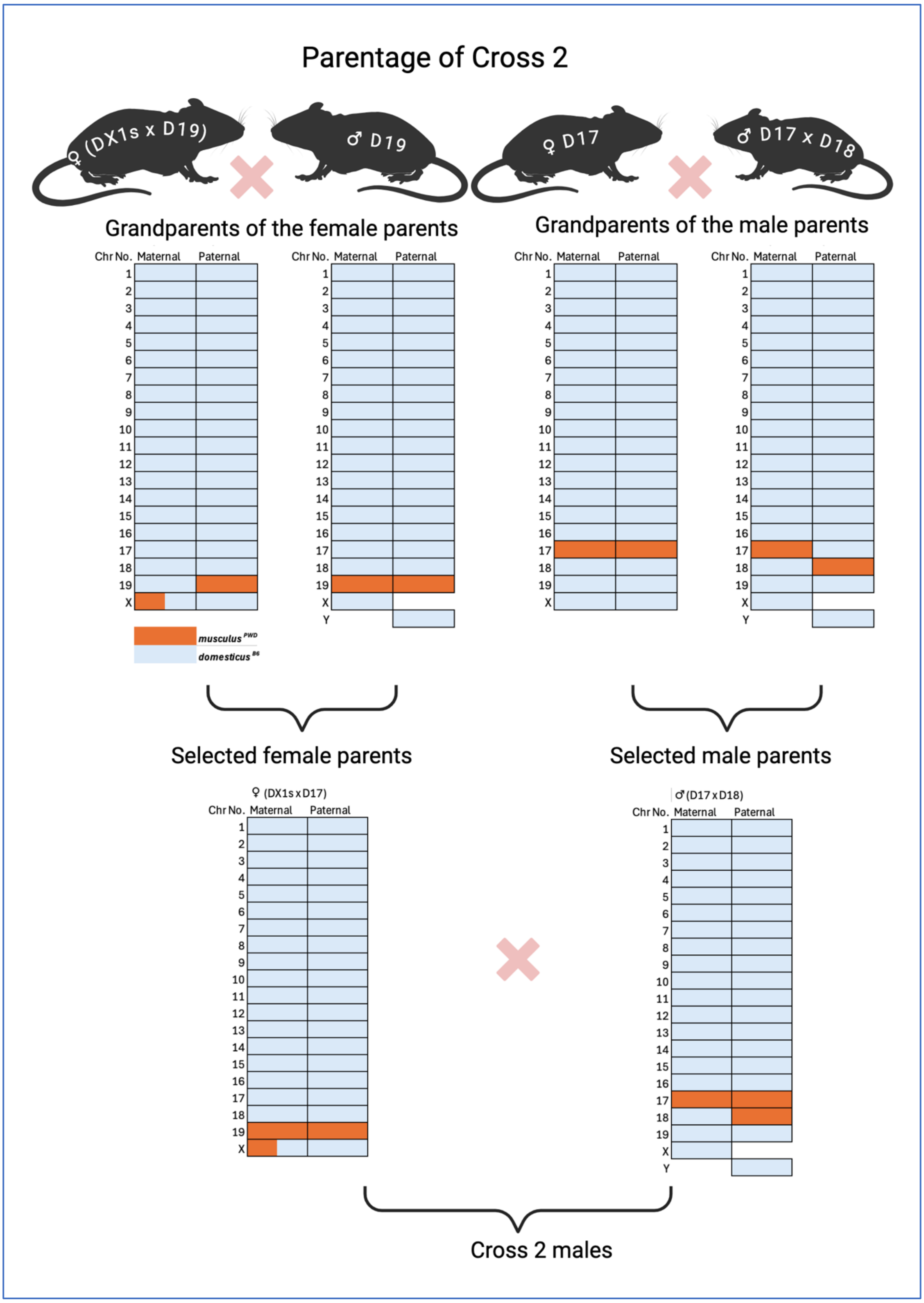

Fig. S2 | Parentage of Cross 2. The chromosomal constitutions of the consomic grandparents of the female and male parents of cross 2 are shown. The female parents were selected to carry the nonrecombinant PP Chr 19 and DX1s Chr X.

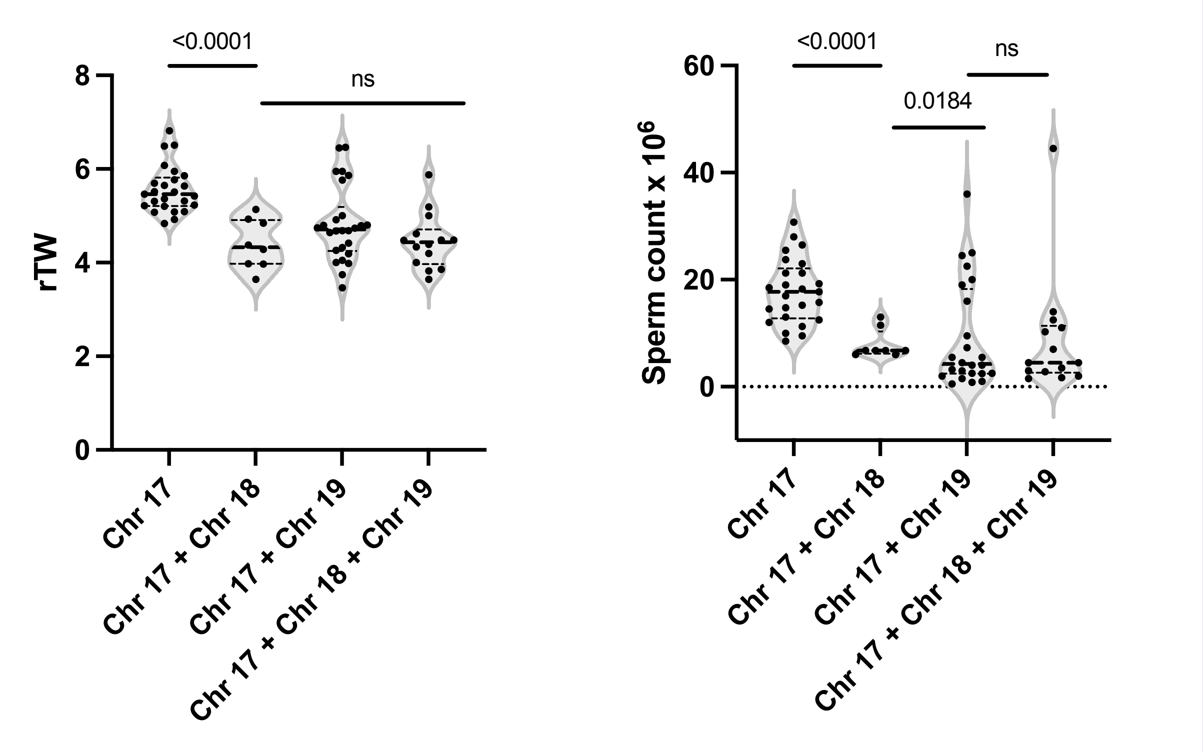

Fig. S3| Comparison of the increasing load of nonrecombinant PWD chromosomes in the B6 genome on rTW and sperm count. Data on Chr 17 and Chrs 17 and 18 are from Cross 1 and data on Chrs 17 and 19 and Chrs 17, 18 and 19 are from Cross 2

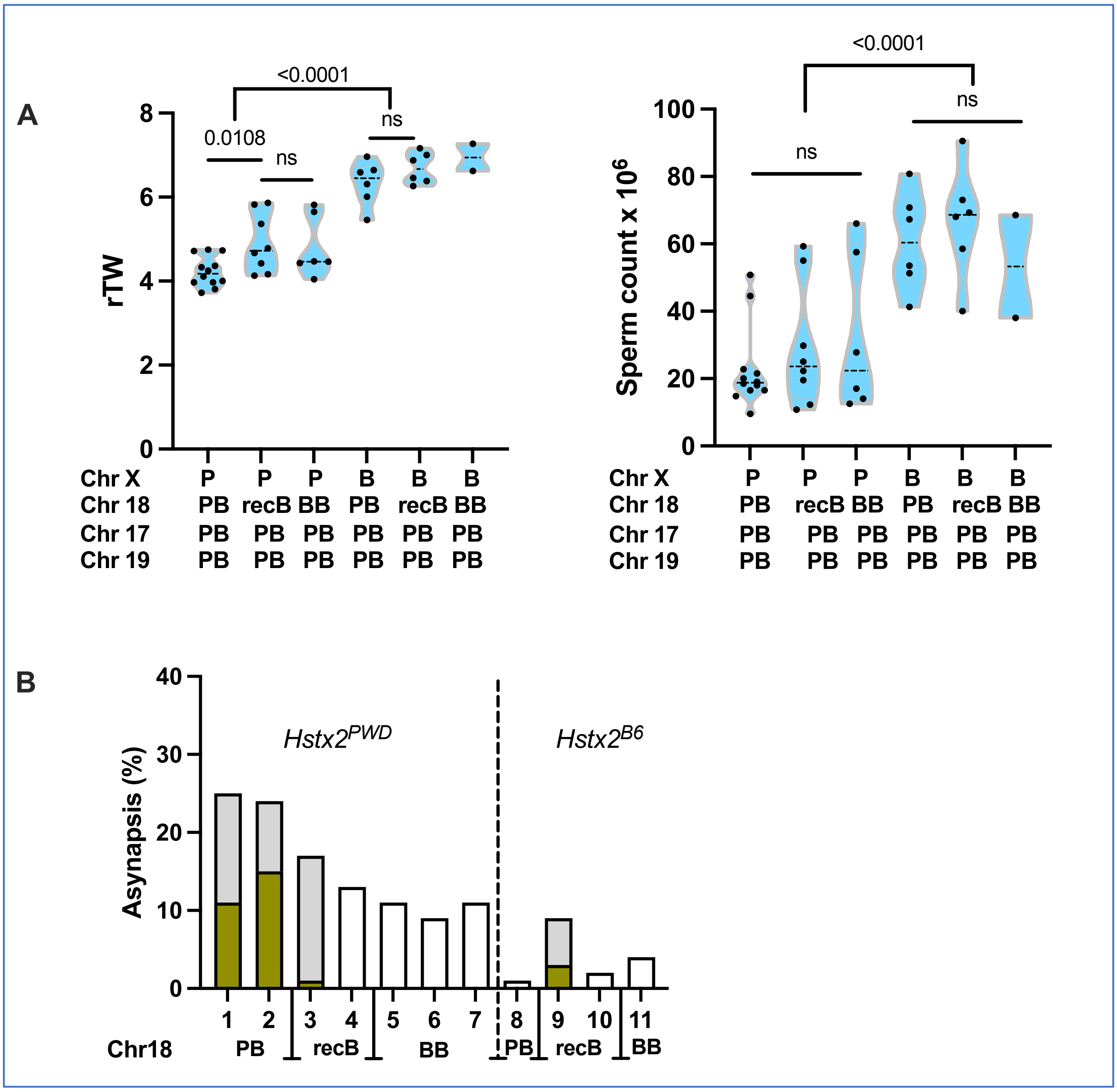

Fig. S4| Fertility parameters (***A***) and asynapsis rate (***B***) in eleven aged Cross 2 males. The effect of the nonrecombinant PB Chr 18 on fertility phenotypes disappeared in the presence of the Chr 19 nonrecombinant PB pair, but the effect of the *Hstx2*^{B6} allele on fertility and asynapsis persists. Green column – frequency of pachynemas with asynapsed Chr 18; grey column – frequency of pachynemas with asynapsed chromosomes other than Chr 18; white column – frequency of pachynemas with any autosomes asynapsed in males without Chr 18 FISH.

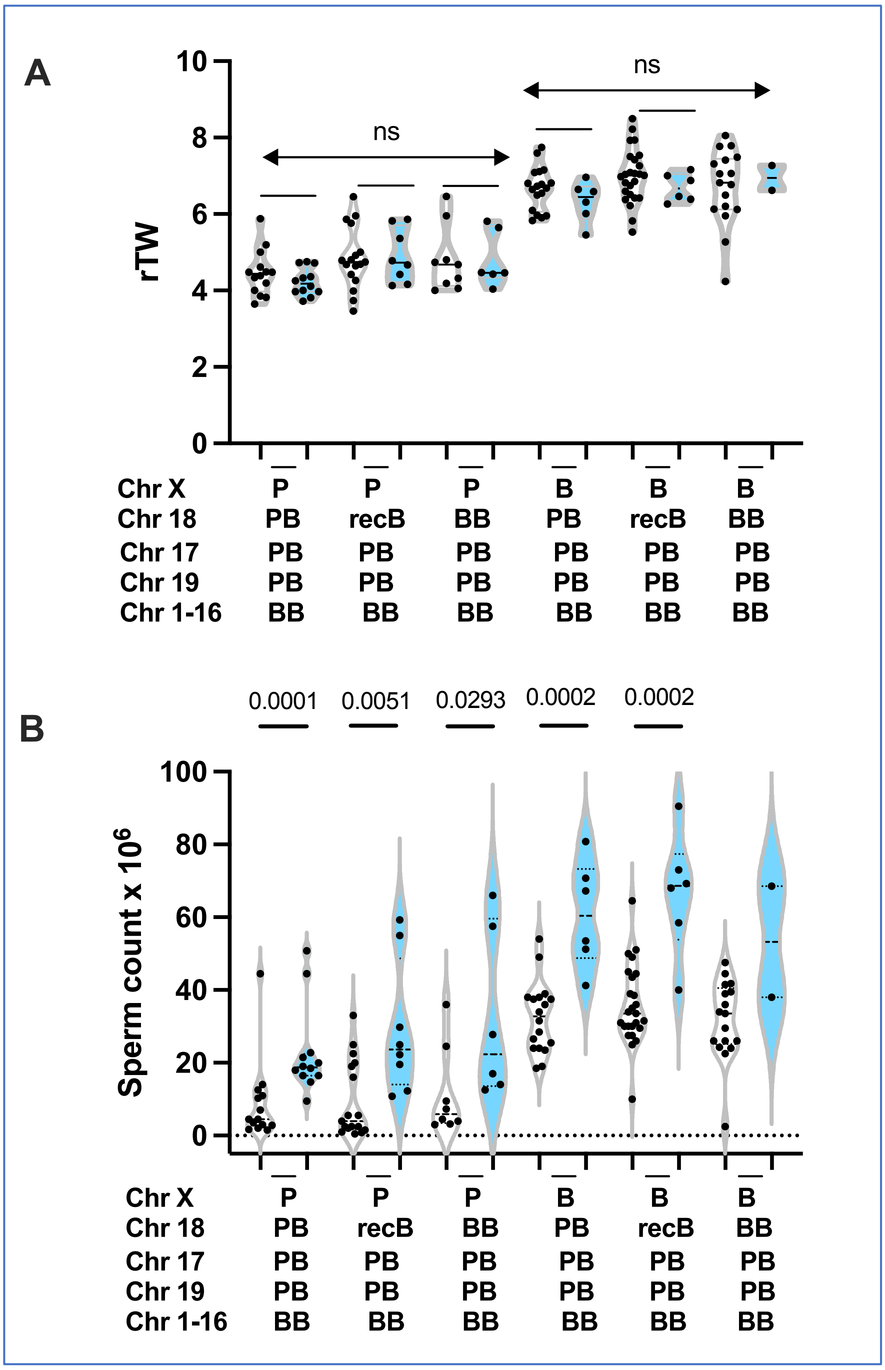

Fig. S5| The effect of aging on the fertility phenotypes of Cross 2 males. ***A.*** rTW does not change with age in any of the six genotypes, in contrast to the significant increase in sperm count (***B***) in Cross 2^{aged} males. Aged Cross 2 males are in blue violin plots.

Supplementary Tables

Table S1 | Cross 1 experiment. The males are sorted by their DXSR51 marker on Chr X (P = PWD allele, B = B6 allele) followed by Chr 18 genotype (PB = PWD, B = B6; BW body weight, rTW relative weight of paired testes, SC sperm count, Log10(1+SC) normalized SC. See Material and Methods for details.

Table S2 | Cross 2 experiment. The males are sorted by their DXSR51 marker on Chr X (P = PWD allele, B = B6 allele) followed by Chr 18 genotype (PB = PWD, B = B6; | = recombination interval). BW body weight, rTW relative weight of paired testes, SC sperm count, Log10(1+SC) normalized SC. See Material and Methods for details.

Table S3 | Cross 2^{aged} experiment. The males are sorted by their DXSR51 marker on Chr X (P = PWD allele, B = B6 allele) followed by Chr 18 genotype (PB = PWD, B = B6; BW body weight, rTW relative weight of paired testes, SC sperm count, Log10(1+SC) normalized SC. See Material and Methods for details.

Table S4 | List of protein coding and RNA genes within the 7.3 Mb interval of the *Hstx3* locus (Mouse Genome Informatics, Ensembl annotation of GRCm39).

Table S5| Whole genome genotyping of over 3500 SNPs in 15 randomly selected males from all three crosses using MiniMUGA arrays.

Table S6| List of primers used for genotyping Chr 17, Chr 18, Chr 19 and Chr X.
